# Proceedings of the Austin District Medical Society

**Published:** 1888-07

**Authors:** T. J. Bennett

**Affiliations:** Secretary


### PROCEEDINGS OF THE AUSTIN DISTRICT MEDICAL SOCIETY.

The Fourth Quarterly Meeting of the Austin District Medical Society was held in Medical Hall, in the city of Austin, on the 14th day of June, 1888.

Dr. Thos. D. Wooten, President, called the society to order at 10 o'clock a. m.

After disposing of the minutes of the last meeting, the following gentlemen were elected to membership: Drs. W. P. Flemming, of Georgetown; G. B. Underhill, of New Braunfels; J. R. Reeve, of Johnson City, Blanco county; B. B. Church, of Austin; J. A. Davis, of Lunatic Asylum, Austin, and H. Leonards, of New Braunfels.

The Secretary announced that several letters from prominent members had been received, expressing regrets at not being able to attend.

Dr. F. R. Martin, of Kyle, who was detained at home on account of a severe attack of conjunctivitis, sent in an interesting report of three cases of poisoning ; one with thirty grains of sulphate copper and three grains of sulphate strychnia ; another with coal oil, and the other with buck-eye, a familiar red berry which grows on a small tree in the country. All three cases recovered. The report was read by J. D. Underhill and referred to the Publishing Committee.

In discussing the cases reported by Dr. Martin, Dr. Morris said his observations concerning the effects of buckeye coincided with those expressed by Dr. Martin, that the symptoms were nearly identical with those of stramonium and bella donna poisoning ; and that the indications for treatment were practically the same.

Dr. Wooten remarked that he was always interested in the reports of such cases, because they are emergency cases to which every physician is liable to be called, and he should know how to meet snch emergies. To him it was unaccountable how the patient could take thirty grains of sulphate copper without its producing emesis and thereby ejecting the three grains of strychnia.

Dr. Denton, referring to the case poisoned by coal oil, stated that he had seen a number of such cases among the insane where the drug was taken with suicidal extent. In every case the symptoms were similar to turpentine poisoning—profound intoxication, strangury, etc. He did not know how much kerosine was taken in each case, but in one he thought more than a pint had been swallowed.

Dr. Bennett read the next paper entitled : "*Hemorrhoids, Fissures and Fistula in Ano, and their Treatment.*"

In the treatment of hemorrhoidal tumors, the following plan was advocated : First divulse the splincter and then catch up the tumors with a pair of dressing forceps and then break them down roughly with a metacarpal saw or the point of a scalpel. The aim is to make a contused or lacerated wound, in order to prevent undue hemorrhage. The paper being upon a most practical subject, was discussed at some length by Drs. Sam Cunningham, Underhill, Denton, Wooten, Swearingen, Tyner and McLaughlin.

AFTERNOON SESSION, 3 O'CLOCK.

Dr. J. W. McLaughlin read a paper entitled: "*The Pathology and Treatment of Chronic Endo-cervicitis.*"

The paper was carefully written and in the main was a concise resume of the Apostoli method of treating uterine disease by electricity.

A very lively discussion followed, the bent of which was against electricity as a remedial agent as now understood and practiced. Dr. Swearingen, Denton, Wooten, Cock and W. M. Cunningham engaged in the discussion.

Dr. G. B. Underhill read a very scientific paper entitled: "*The Influence of Malarial Poison Upon the Upper Abdominal Viscera, Especially Upon the Spleen.*"

The author attempted to account for the various phenomena observed in malarial disease by the action of the poison upon the ganglionic system of the nerves. This paper will be read with much interest by physicians in malarial districts. It was especially complimented.

Dr. Sam Cunningham read a voluntary report of a "*Case of Plcenta Prævia with Twins.—Recovery.*"

Remarks were made upon this paper by Drs. Bennett, Denton, Swearingen, W. M. Cunningham and Wooten.

Under the head of reports of cases Dr. Wooten stated that he had been called that morning to a child about six years of age with a bean lodged somewhere in the left bronchus. The bean had been in this position three or four days before he saw the patient. There was broncho-pneumonia, as was evidenced by considerable dullness over all the upper portion of the left lung, and the terperature at 102° F.

The question was asked: "What shall be done? Operate or adopt the more conservative course?" The latter advice prevailed.

Dr. A. N. Denton read a paper on "*The Management of the Insane,*" it being a continuation of a paper read on the same subject at the last meeting of the society.

The paper was discussed by Drs. Preston, Swearingen and McLaughlin.

Dr. Denton was requested by the Society to continue the subject at the September meeting.

The papers were all referred to the Publishing Committee and will appear in the JOURNAL in the near future.

[It is to be regretted that the discussions were not reported, since they would have been of greater value to the profession than the papers. Discussion gives the opinion of many instead of one.—ED.]

A committee, consisting of Drs. Swearingen and Bennett was appointed, to act with the committee from the State Association, to secure rooms for a Medical Museum and Library in the new capitol.

The Society expressed regrets at Dr. Daniel's not being able to attend the meeting, but congratulated him upon getting the upper hand of an attack of cholera.

There being no other business, the Society adjourned after a most pleasurable and profitable day's work, to meet again in Austin in September next.

T. J. BENNETT, Secretary.

---

